# Teleconsultation Between Patients and Health Care Professionals in the Catalan Primary Care Service: Message Annotation Analysis in a Retrospective Cross-Sectional Study

**DOI:** 10.2196/19149

**Published:** 2020-09-17

**Authors:** Francesc López Seguí, Sandra Walsh, Oscar Solans, Cristina Adroher Mas, Gabriela Ferraro, Anna García-Altés, Francesc García Cuyàs, Luis Salvador Carulla, Marta Sagarra Castro, Josep Vidal-Alaball

**Affiliations:** 1 TIC Salut Social Ministry of Health Barcelona Spain; 2 Centre de Recerca en Economía de la Salut Pompeu Fabra University Barcelona Spain; 3 Institut de Biologia Evolutiva (CSIC) Pompeu Fabra University Barcelona Spain; 4 Health Department Catalan Ministry of Health Barcelona Spain; 5 Sant Joan de Déu Hospital Barcelona Spain; 6 CSIRO Australian National University Canberra Australia; 7 Agency for Healthcare Quality and Evaluation of Catalonia Catalan Ministry of Health Barcelona Spain; 8 Research School of Population Health Australian National University Canberra Australia; 9 Centre d’Atenció Primària Capellades Gerència Territorial de la Catalunya Central Institut Català de la Salut Barcelona Spain; 10 Health Promotion in Rural Areas Research Group, Gerència Territorial de la Catalunya Central Institut Català de la Salut Sant Fruitós de Bages Spain; 11 Unitat de Suport a la Recerca de la Catalunya Central Fundacio Institut Universitari per a la recerca a l’Atencio Primaria de Salut Jordi Gol i Gurina Sant Fruitós de Bages Spain

**Keywords:** teleconsultation, primary care, remote consultation, message annotation, face-to-face visits

## Abstract

**Background:**

Over the last decade, telemedicine services have been introduced in the public health care systems of several industrialized countries. In Catalonia, the use of eConsulta, an asynchronous teleconsultation service between primary care professionals and citizens in the public health care system, has already reached 1 million cases. Before the COVID-19 pandemic, the use of eConsulta was growing at a monthly rate of 7%, and the growth has been exponential from March 15, 2020 to the present day. Despite its widespread usage, there is little qualitative evidence describing how this tool is used.

**Objective:**

The aim of this study was to annotate a random sample of teleconsultations from eConsulta, and to evaluate the level of agreement between health care professionals with respect to the annotation.

**Methods:**

Twenty general practitioners retrospectively annotated a random sample of 5382 cases managed by eConsulta according to three aspects: the type of interaction according to 6 author-proposed categories, whether the practitioners believed a face-to-face visit was avoided, and whether they believed the patient would have requested a face-to-face visit had eConsulta not been available. A total of 1217 cases were classified three times by three different professionals to assess the degree of consensus among them.

**Results:**

The general practitioners considered that 79.60% (4284/5382) of the teleconsultations resulted in avoiding a face-to-face visit, and considered that 64.96% (3496/5382) of the time, the patient would have made a face-to-face visit in the absence of a service like eConsulta. The most frequent uses were for management of test results (26.77%, 1433/5354), management of repeat prescriptions (24.30%, 1301/5354), and medical enquiries (14.23%, 762/5354). The degree of agreement among professionals as to the annotations was mixed, with the highest consensus demonstrated for the question “Has the online consultation avoided a face-to-face visit?” (3/3 professionals agreed 67.95% of the time, 827/1217), and the lowest consensus for the type of use of the teleconsultation (3/3 professionals agreed 57.60% of the time, 701/1217).

**Conclusions:**

This study shows the ability of eConsulta to reduce the number of face-to-face visits for 55% (79% × 65%) to 79% of cases. In comparison to previous research, these results are slightly more pessimistic, although the rates are still high and in line with administrative data proxies, showing that 84% of patients using teleconsultations do not make an in-person appointment in the following 3 months. With respect to the type of consultation performed, our results are similar to the existing literature, thus providing robust support for eConsulta’s usage. The mixed degree of consensus among professionals implies that results derived from artificial intelligence tools such as message classification algorithms should be interpreted in light of these shortcomings.

## Introduction

### Five Years of Teleconsultation in the Catalan Public Health System

The Catalan health care System dispenses services for 7.6 million inhabitants, providing universal coverage through a tax-based system. Administratively, it is composed of a single public payer and multiple service providers that are publicly or privately owned, with an integrated system, a major role in community and primary health care, and the use of information technologies and digital health [1].

The Catalan Health Institute (Institut Catala de la Salut) is the provider of primary health care services to three-quarters of the population of Catalonia [2]. Over the last decade, telemedicine services have been introduced in the public health care systems of several industrialized countries. In Catalonia, eConsulta is an asynchronous teleconsultation service that has been integrated into the program of computerized medical records of primary care of the public health system [3]. The service was introduced in 2015 and was gradually phased in until 2017, when it became established as a service available to all primary care teams as a complement to face-to-face care. Although initially intended as a tool solely for general practitioners, eConsulta can now also be used by pediatricians, gynecologists, midwives, and nurses. At present, 94.9% (353/372) of primary care teams have used the tool, although this type of consultation still accounts for less than 1% of the total in primary care [4]. From the patients’ point of view, eConsulta is one of the services forming part of their personal health folder, and is considered to be a tool that allows the user to access aspects of their health information, make enquiries, and carry out certain administrative procedures. In this space, which is accessed via a secure authentication process, the eConsulta interface allows users to choose which health professional to direct their enquiry to and attach files, while keeping a record of previous interactions.

**Figure 1:**
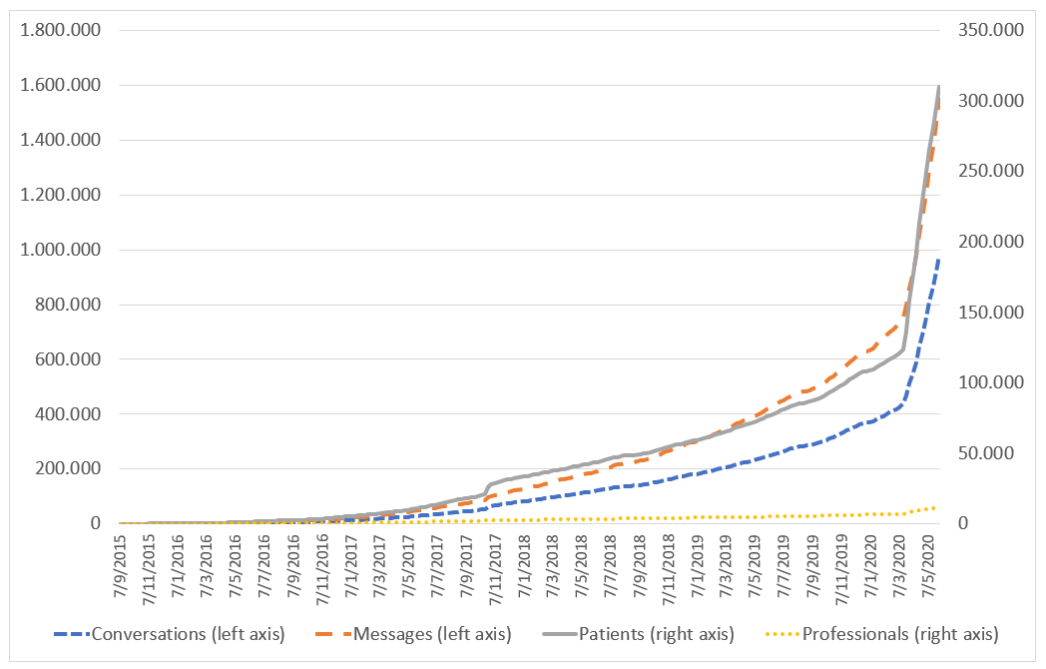
Numbers of messages and conversations (left axis), and patients and health care professional users (right axis) of eConsulta.

**Figure 2:**
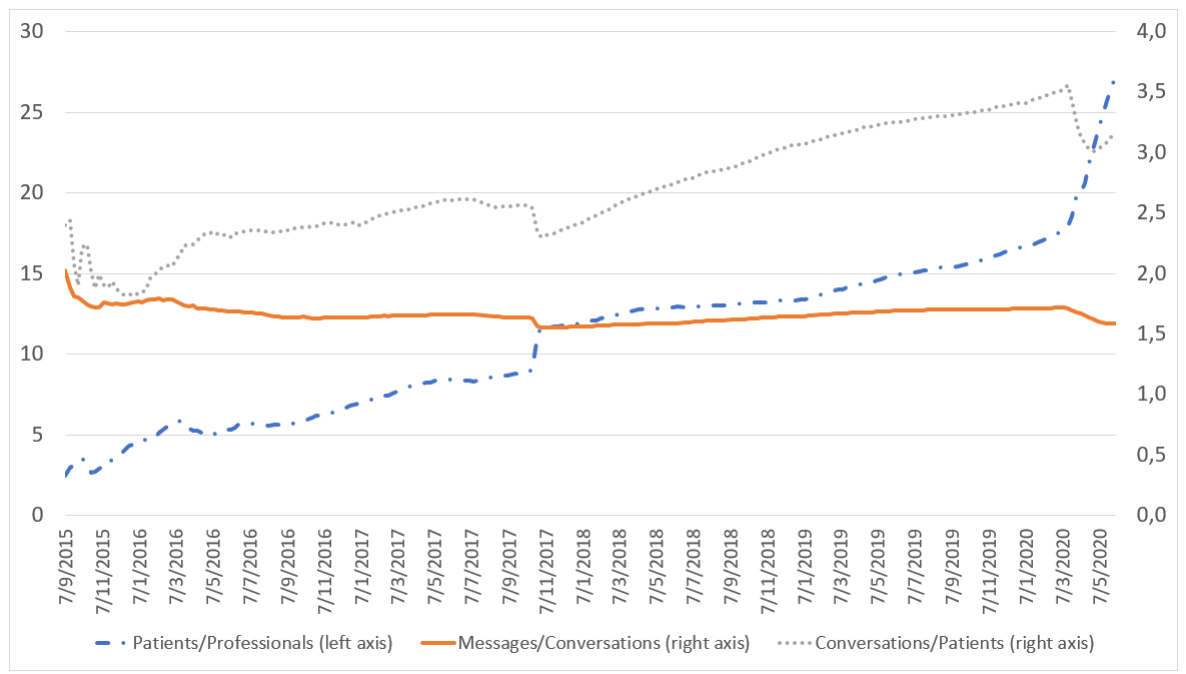
Patients per professional (left axis), messages per conversation and conversation per patient (right axis) in eConsulta.

As of June 8, 2020, eConsulta collected 1,630,881 messages corresponding to 1,030,536 conversations between 322,897 unique users and 11,620 primary care professionals (59% doctors, 41% nurses). Before the COVID-19 pandemic, eConsulta was already growing at a rate of 24,000 conversations, 44,000 messages, 5500 new users, and 140 new professionals per month, representing a monthly growth rate of 7%, 6%, 5%, and 2%, respectively. These rates have increased exponentially from March 15, 2020 to the present day (Figure 1). The impact of the pandemic on the Catalan digital health ecosystem has already been analyzed in a previous study [5], demonstrating that the average number of messages per conversation has been stable at 1.71, suggesting that many conversations contain a single message (Figure 2). Professionals and users have used the tool an average of 17.18 and 3.47 times, respectively. However, these figures continue to grow, indicating that users are satisfied with the experience.

In terms of the content and impact of eConsulta on face-to-face visits, a previous study [6] suggested that there was broad consensus among general practitioners that eConsulta has potential to resolve patient queries (avoiding the need for a face-to-face visit in 88% of cases), but that it induced demand (queries which otherwise would not have been made) in 28% of cases, and the most common use was for the management of test results (35%), clinical enquiries (16%), and the management of repeat prescriptions (12%). However, this study was based on a very small sample of cases involving general practitioners who use the tool in a semirural geographical area. Another study analyzed the predictive ability of machine-learning algorithms to identify types of conversations based on body text, but obtained very few conclusive results [7]. Understanding the nature of the interaction (ie, the main topics of conversation) can help to manage such an online health care consultation tool more effectively and efficiently, which is essential considering the rapid rate at which its usage is growing.

### Study Aim

The aim of the present study was to classify a random sample of teleconsultations on eConsulta according to three types of information: the type of consultation based on 6 categories, professionals’ judgement on whether or not a face-to-face visit was avoided, and whether professionals believe that the patient would have requested a face-to-face visit had eConsulta not been available. As a secondary objective, we evaluated the level of agreement among the health care professionals with respect to their annotation.

## Methods

### Sample

**Figure 3:**
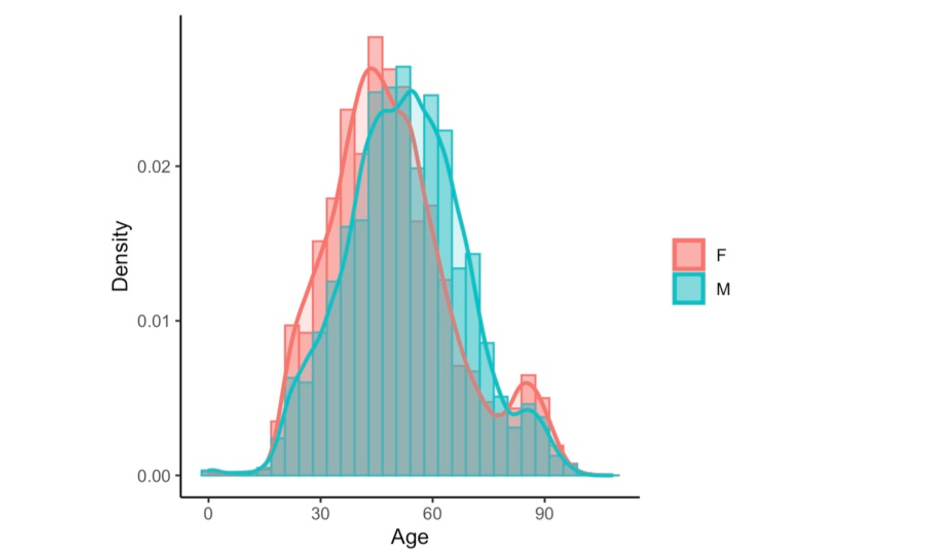
Age profile of patient users of the teleconsultation service, according to gender.

The sample analyzed included teleconsultations involving Catalan Health Institute centers initiated before September 22, 2019, comprising 403,274 messages, 236,178 conversations, and 69,111 unique users from all over Catalonia. Among the total conversations, 71.46% (168,772/236,178) were initiated by the patient, with the others initiated by the health care professional. In addition, 84.88% (187,569/220,981) of the conversations had a general practitioner as the clinical interlocutor, with the others being nurses (14.31%, 31,622/220,981) or other professionals such as pediatricians and gynecologists. The characteristics of professionals that use eConsulta were previously reported, demonstrating an age range of the general practitioners of 45-54 years, who scored higher than the 80th percentile on the quality of care index, had a high degree of accessibility, involved in teaching, and work on a health team in a high socioeconomic urban setting [3]. Messages and their respective subject lines had an average length of 231 and 18 characters, respectively. The users of the service were mostly women (57.00%, 39,393/69,111) and their average age was 50.62 years (SD 16.59) (Figure 3).

**Figure 4:**
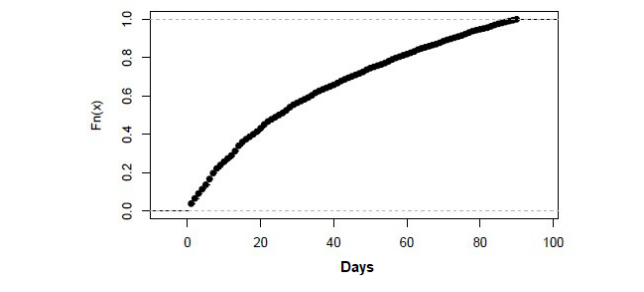
Days until face-to-face visit for eConsultations that resulted in a face-to-face visit in the following 90 days (accumulated density).

With regard to the use of face-to-face resources of the sample analyzed, the date of the next face-to-face visit and the total number of visits registered to the primary care team during the current year are available. Since the teleconsultation, 72.21% (150,135/207,910) of the cases did not subsequently visit the primary care team for a face-to-face consultation (for any reason), whereas 15.78% (32,802/207,910) had a consultation within the following 3 months and 12.01% (24,973/207,910) did so after 90 days. Assuming that visits made after 3 months may not be associated with the reason for the initial teleconsultation, 84.22% (175,108/207,910) of the online consultations may not be related to a subsequent face-to-face visit, suggesting that it is an effective tool for avoiding face-to-face visits. These results are in line with a previous study [6] and did not depend on the initiator of the conversation (Figure 4). Moreover, among users of teleconsultations who recorded at least one face-to-face visit, use of the in-person service occurred an average of 2.3 times a year.

Finally, in most of the online consultations that were associated with a subsequent face-to-face visit, the visit took place within the first 20 days after the teleconsultation, indicating the degree of accessibility to treat the patient in person.

### Annotation Procedure

A random sample of 5460 unique conversations on eConsulta was generated, 4200 of which were selected to be annotated only once and the remaining 1260 were selected to be annotated three times by each general practitioner to evaluate the degree of consensus among professionals. Thus, a total of 7980 (4200+1260×3) conversations were randomly distributed among 20 health care professionals linked to the eConsulta Research Group (eHealth Office, Catalan Ministry of Health). The text in the subject line and message body was analyzed once the data were anonymized. Following the methods of López et al [6], each general practitioner recorded three pieces of information for each of their interactions: the type of consultation according to 6 author-proposed categories (see Multimedia Appendix 1), which is a modified version of our previously used classification system [6]; whether they believed a face-to-face visit was avoided, which was defined as the absence of the need for a face-to-face visit following the consultation; and whether they believed the patient would have requested a face-to-face visit had eConsulta not been available. The first aspect is an indicator of the effectiveness of the intervention, whereas the latter two questions were used as an approximate measure of the possible increased demand resulting from the ease of access to a general practitioner.

When agreeing to participate in the analysis, the participating health care professionals received a guide to standardize the annotation criteria as much as possible. The annotators were unaware that some of the conversations were being annotated three times and that the consensus among them was going to be evaluated. The study was approved by the Ethical Committee for Clinical Research at the Foundation University Institute for Primary Health Care Research Jordi Gol i Gurina (registration no. P19/096-P).

### Data Analysis

The raw data were processed and analyzed with Bash and R languages (using Rstudio version 1.2.1335 and R version 3.5.3) with the packages data.table, dplyr, and ggplot2.

## Results

### Annotation

Overall, 98.57% (5382/5460) of the unique conversations in the sample were classified. Among these, for the subset of messages that were annotated three times (22.61%, 1217/5382), the annotation with the highest consensus was selected. Table 1 shows the results of the three main annotated variables.

**Table 1:** Results of the annotation.

Variable		n (%)
**Has the online consultation avoided a face-to-face visit? (N=5382)**		
	Yes	4284 (79.60)
	No	740 (13.75)
	Not sure	360 (6.69)
**In the absence of a service like eConsulta, would the patient have had a face-to-face consultation? (N=5382)**		
	Yes	3496 (64.96)
	No	1626 (30.21)
	Not sure	260 (4.83)
**Type of teleconsultation (N=5354)**		
	Management of test results	1433 (26.77)
	Temporary disability management	299 (5.58)
	Management of visits/referrals	536 (10.01)
	Repeat prescriptions	1301 (24.30)
	Medical enquiries	762 (14.23)
	Other	1023 (19.11)

According to the health professionals’ annotation, the majority of the teleconsultations would have avoided a face-to-face visit. In addition, in the absence of the service, the patient would have arranged a face-to-face consultation in most cases (Table 1). Specifically, this study shows that eConsulta has the ability to decrease the number of face-to-face visit in between 55% (79%×65%) and 79% of cases. The most frequent uses were for the management of test results, the management of repeat prescriptions, and for general medical enquiries (Table 1).

Table 2 shows the results of the response to the questions “Has the online consultation avoided a face-to-face visit?” and “In the absence of a service like eConsulta, would the patient have had a face-to-face consultation?” according to the type of consultation. The ability of the teleconsultation to avoid a face-to-face visit was relatively higher for consultations related to the management of test results and repeat prescriptions, as the most frequent types of consultation, which are also those with lower induced demand. This implies that health professionals are using the tool in the most effective circumstances.

**Table 2:** Relationship between the type of consultation and the two other variables recorded.

Type of teleconsultation	N	Has the online consultation avoided a face-to-face visit? n (%)				In the absence of a service like eConsulta, would the patient have had a face-to-face consultation? n (%)		
		Yes	No	Not sure	Yes		No	Not sure
Management of test results	1433	1319 (92.04)	71 (4.95)	43 (3.00)	1105 (77.11)		323 (22.54)	5 (0.35)
Temporary disability management	299	265 (88.6)	27 (9.0)	7 (2.3)	217 (72.6)		82 (27.4)	0 (0.0)
Management of visits/referrals	536	397 (74.1)	120 (22.4)	19 (3.5)	341 (63.6)		187 (34.9)	8 (1.5)
Repeat prescriptions	1301	1223 (94.00)	50 (3.84)	28 (2.15)	988 (75.94)		311 (23.90)	2 (0.15)
Medical enquiries	762	586 (76.9)	146 (19.2)	30 (3.9)	520 (68.24)		226 (29.7)	16 (2.1)
Other	834^a^	468 (56.1)	315 (37.8)	51 (6.1)	311 (37.3)		488 (58.5)	35 (4.2)

^a^Although the total N of the “Other” category was 1023, only 834 observations were classified into the subcategories for these two variables.

### Consensus Among Professionals

Table 3 shows the degree of consensus among professionals for the 1217 conversations that were annotated three times (96.58% of the target of 1260), according to three levels for each of the variables. Level 1 indicates that all professionals agree on the annotation, level 2 indicates that two of the three professionals agree on the annotation, and level 3 indicates that all three professionals responded differently to the same annotation.

**Table 3:** Consensus among three annotators for annotated conversations (N=1217).

Annotated variables	Level 1 (3/3 agreement), n (%)	Level 2 (2/3 agreement), n (%)	Level 3 (no agreement), n (%)
Has the online consultation avoided a face-to-face visit? (Yes/No/Not sure)	827 (67.95)	356 (29.25)	34 (2.79)
In the absence of a service like eConsulta, would the patient have had a face-to-face consultation? (Yes/No/Not sure)	477 (39.19)	713 (58.59)	27 (2.21)
Type of teleconsultation (Categories 1-6^a^/Not sure)	701 (57.60)	438 (35.99)	78 (6.41)

^a^See Multimedia Appendix 1 for category numbers and descriptions.

Notably, the lowest level of agreement (level 3) was negligible for all three variables. Responses to the question on whether eConsulta avoided a face-to-face visit generated the highest consensus, whereas the responses to whether eConsulta resulted in an induced demand generated the least consensus. The latter can be attributed to confusion as to the annotation process. Finally, it is necessary to keep in mind that the variable “type of teleconsultation” has more possible answers than offered; thus, absolute consensus among annotators (3/3) is less likely. Nevertheless, in 93.6% (1139/11217) of cases, at least 2/3 of the annotators coincided at the time of classifying the query.

## Discussion

### Principal Findings

This study focused on the use of teleconsultation in primary care in the Catalan health system by recruiting 20 health care professionals to annotate a total of 5382 cases. In line with the results of an earlier study [6], we found that eConsulta is a tool that primary care professionals mainly use to inform patients of their test results and for aspects related to the medication plan (eg, repeat prescription, expiration), for which it shows a high rate of resolution (avoiding a face-to-face visit). “Crossvalidation” among the professionals’ annotations showed a high degree of consensus.

A major use of eConsulta appears to be for administrative or logistical reasons. This suggests that if the type of task to be performed by each professional was reformulated, some queries could be directed to nursing or even administrative staff. Given the growing pressures on primary care in Catalonia and the difficulty in recruiting general practitioners and pediatricians in some areas of the country, eConsulta could serve as a tool to empower other professional profiles in the management of certain cases. Although the results showed that health care professionals are able to resolve such cases efficiently through the use of this tool, freeing them from tasks that have little clinical value could increase their satisfaction and boost the efficiency of the primary health care system. Despite the relatively high rate (76%), medical enquiry–based consultations showed reduced potential of online resolution compared to other type of consultations, suggesting that policymakers should be cautious about the use of this kind of telemedicine service for this specific type of consultation. The types of consultations that are more likely to have increased demand should also be taken into account so as to control the amount of queries that might be received in the teleconsultation service.

From the user’s point of view, the high virtual resolution of the queries also suggests a possible increase in their satisfaction [8,9]. The present results show that certain queries that are made telematically would not have been made in the absence of this type of service, thus improving people’s access to the health care system and empowering them in the management of their own health and care.

For future developments aimed at increasing the capacity and optimizing the use of eConsulta, the reasons for the interactions should be systematically parameterized, which can facilitate the use of the system by the public and the development of apps based on trustable, transparent, and interpretable artificial intelligence. Simultaneously, each case should be directed to the most appropriate professional to obtain a response, leading to better case management and more efficient use of resources from the first interaction, while generating good habits in the relationship between the patient and the health care professional.

To conclude, it should be mentioned that if virtual consultations are to be used more frequently as a tool for clinical communication between health professionals and patients for health care reasons, other synchronous communication tools such as video conferencing ought to be developed [10]. These additional tools could be useful for patients with mild acute pathologies as well as for patients with chronic diseases with a high need for follow up, which can help to avoid successive face-to-face visits and improve the continuity of care [11].

### Limitations

The high monthly growth of new users suggests that there are many first conversations, implying that there could be a large number of experimental queries. This would explain the fact that some of the conversations were annotated in the “Other” category. However, the fact that information systems do not record the reason for the teleconsultation makes it very difficult to analyze their association with the use of face-to-face resources.

Finally, subsequent studies should further confirm the degree of consensus with respect to the question “In the absence of a service like eConsulta, would the patient have had a face-to-face consultation?”

## Appendix

Multimedia Appendix 1 Categories of patient-physician consultation types.
