# Supplementary material for: Teleconsultation Between Patients and Health Care Professionals in the Catalan Primary Care Service: Message Annotation Analysis in a Retrospective Cross-Sectional Study
Source: J Med Internet Res. 2020 Sep 17;22(9):e19149. doi: 10.2196/19149 (PMC7530682; doi:10.2196/19149)
Supplement: Multimedia Appendix 1 [file jmir_v22i9e19149_app1.docx]

**Reasons for patients and general practitioners to use eConsulta**

1. Management of test results

- The patient provides the results of tests from an external centre so they are recorded in their medical history.
- The GP provides normal test results.
- The GP deals with test-related questions from the patient.
- The GP requests tests after conducting a follow-up teleconsultation.

2. Temporary disability management

- The patient communicates changes to their health related to an upcoming temporary disability.
- The GP tracks the progress of a temporary disability in conjunction with face-to-face visits.

3. Management of visits/referrals

- The patient has an enquiry which the GP thinks ought to be dealt with by a specialist and refers them. They can also report incidents resulting from any referrals made.
- The GP resolves incidents relating to the timing of visits.
- The GP cancels visits from other clinicians in cases in which the problem has been resolved following completion of the eConsulta.
- Validation of appointments with other specialists where the patient needs more information regarding the motivation for the appointment.

4. Repeat prescriptions

- The patient asks for their prescription to be updated if it has been modified by an external specialist, either because they do not use it or because it has expired.
- The GP warns the patient that their prescription is about to expire and updates it.
- The GP cancels an unnecessary prescription following an eConsulta.

5. Medical enquiries: the patient has a question about their health that can be resolved without a physical examination. They can also attach photographs to accompany the description.

6. Other: any other type not covered by this classification.
